# The Molecular Mechanism of Sex Hormones on Sertoli Cell Development and Proliferation

**DOI:** 10.3389/fendo.2021.648141

**Published:** 2021-07-23

**Authors:** Wasim Shah, Ranjha Khan, Basit Shah, Asad Khan, Sobia Dil, Wei Liu, Jie Wen, Xiaohua Jiang

**Affiliations:** The First Affiliated Hospital of USTC, Division of Life Sciences and Medicine, University of Science and Technology of China, Hefei, China

**Keywords:** Sertoli cells, fertility, sex hormone, spermatogenesis, testis

## Abstract

Sustaining and maintaining the intricate process of spermatogenesis is liable upon hormones and growth factors acting through endocrine and paracrine pathways. The Sertoli cells (SCs) are the major somatic cells present in the seminiferous tubules and are considered to be the main regulators of spermatogenesis. As each Sertoli cell supports a specific number of germ cells, thus, the final number of Sertoli cells determines the sperm production capacity. Similarly, sex hormones are also major regulators of spermatogenesis and they can determine the proliferation of Sertoli cells. In the present review, we have critically and comprehensively discussed the role of sex hormones and some other factors that are involved in Sertoli cell proliferation, differentiation and maturation. Furthermore, we have also presented a model of Sertoli cell development based upon the recent advancement in the field of reproduction. Hence, our review article provides a general overview regarding the sex hormonal pathways governing Sertoli cell proliferation and development.

## Background

Testes are destined to perform two important roles: to produce testosterone (steroidogenesis) and to maintain germ cell development (1). These functions are supported by the testicular somatic cells, Sertoli cells (SCs), which are located within the seminiferous tubules of testes (2, 3). Sertoli cells are considered as the most complex type of cells in an organism on the bases of their three-dimensional structure and their production of a microenvironment for germ cell development (3–5). Dependence of germ cells to obtain nutritional contents from Sertoli cells is owing to the presence of blood testes barrier (BTB) which physically portioned the seminiferous tubules into basal and adluminal compartments (6). The BTB is constituted by tight junction, ectoplasmic specialization (N-cadherin), desmosomes and gap junctions that are present in Sertoli cells (7–10). The SC–SC junctional complex has been studied and is known to undertake an indispensable job in testis directional morphogenesis (11, 12). Thus, Sertoli cells encompass all sorts of germ cells and have a chief assistive role in spermatogenesis.

Furthermore, developing germ cells cannot metabolize macromolecules such as lipids, carbohydrates and proteins, and most preferable energy source for germ cells is lactate molecule which is produced by Sertoli cells (13, 14). On the other hand, Sertoli cells not only provide lactate to the developing germ cells for energy production but they also supply other nutrients including amino acids, vitamins and metal ions (14–16). Another important task of Sertoli cells is to generate and produce signaling molecules including growth factors and inflammatory cytokines which are involved in a cascade of events that are necessary for the spermatogenic process (17–19). Thus, accurate establishment and proper functioning of Sertoli cells is crucial for the developing germ cells to sustain the process of spermatogenesis.

## Proliferation and Maturation of Sertoli Cells

The proper proliferation of Sertoli cells takes place during their immature period and can be mediated by specific factors (20, 21). The proliferative phase of Sertoli cells varies between species and two periods of Sertoli cells proliferation (one during fetal or neonatal period and other before pubertal period) are generally observed in various species (4, 6). Furthermore, marked variations exist between mature and immature Sertoli cells especially in terms of morphological and biochemical aspects. Generally, immature Sertoli cells reside on the basement and possess cytoplasmic projections which fill up the space of seminiferous cords (20, 22). In addition to immature Sertoli cells, seminiferous tubules also contain peritubular and germ stem cells which give solid appearance with the absence of lumen (23). After puberty, the Sertoli cells start to elongate and BTB begins to establish (8). Finally, Sertoli cells switch from their immature stage to mature phase and their proliferative state is stopped (6). At this stage, mature Sertoli cells represent radical changes within their morphology and functions. Further changes occurred in the nucleus and nucleoli become large in size along with the completion of tight junction which makes the fluid filled lumen space. The whole process of Sertoli cell proliferation and maturation is regulated under strict control and any impairment in the process of Sertoli cell development or proliferation can causes pathological events which may lead to the reduction of sperm count and semen quality (6, 20, 24–26).

Sertoli cells can serve as the organizing center for testis differentiation and signalings from Sertoli cells also regulate the differentiation events of testicular cord formation and testis organogenesis (5, 27). The Sertoli cells also provide a means of canalizing gonadal fate to coordinate testis development (5). Interestingly, Sertoli cell fate, once specified, is not permanent but instead needs to be constantly reinforced (3, 5).

**Figure 1:**
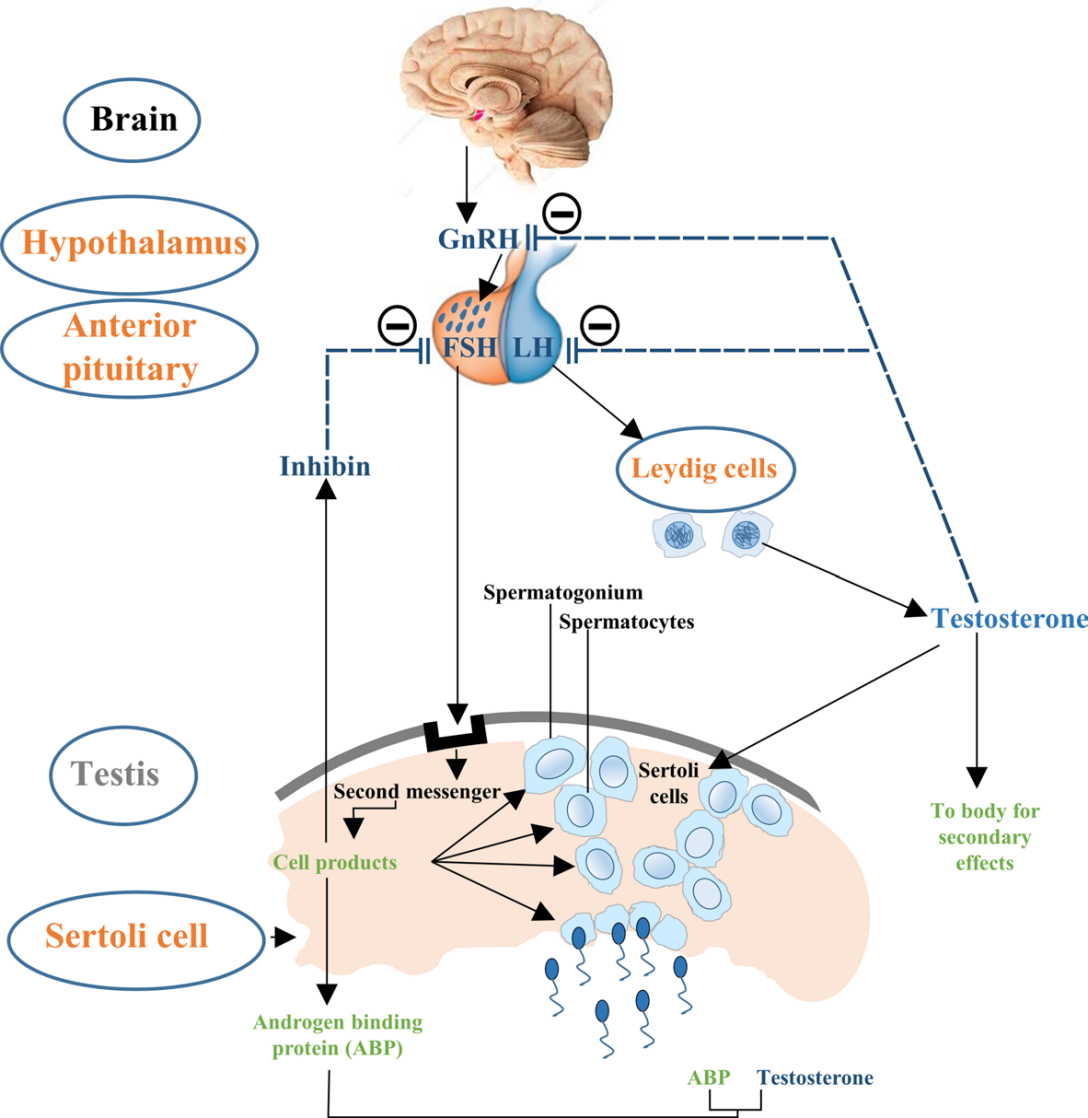
Flow chart description the control of hypothalamus–pituitary–testis axis on Sertoli cell proliferation. The hypothalamic GnRH modulates the biosynthesis and secretion of pituitary hormones i.e., LH and FSH. LH induces secretion of testosterone in Leydig cells and is involved in the late Sertoli cell proliferation period, followed by negative feedback reducing GnRH and LH production. FSH primarily stimulates the seminiferous tubules to form steroid hormones such as inhibin and further sustain the process of spermatogenesis. Steroid hormones i.e., testosterone and inhibin exert negative feedback effects on GnRH.

Testicular development and spermatogenesis are influenced by various hormones which are generally mediated by the hypothalamus–pituitary–gonad (HPG) axis (14, 27) (**Figure 1**). HPG axis establishes a connection between brain and testes (28, 29). The gonadotropin leuitinizing hormone (LH) and follicle stimulating hormone (FSH) are secreted by adenohypophysis which are considered to be the important regulators of testicular function (30). It has been demonstrated that FSH mainly controls the proliferation of Sertoli cells while LH regulates testosterone production (14, 27, 31). Thus, the pre-pubertal decrease of LH and subsequent FSH secretion tends to cause a disturbance in the pulsatile release of gonadotropin-releasing hormone (GnRH). This hypothalamic-releasing agent provides the main push to the gonadotropin-secreting cells of the anterior pituitary gland (32). What is more, the HPG axis also works in association with local endocrine system to mechanistically regulate the complete process of Sertoli cell maturation and testis development (30). Paracrine system intercedes with various types of cells including germ cells, peritubular myoid cells and Leydig cells. Thus, proper hormone levels and their regulation are necessary for these complex processes which further ensure the accurate and smooth development of Sertoli cells to support spermatogenic process.

### Sex Hormones in Sertoli Cell Development and Proliferation

**Figure 2:**
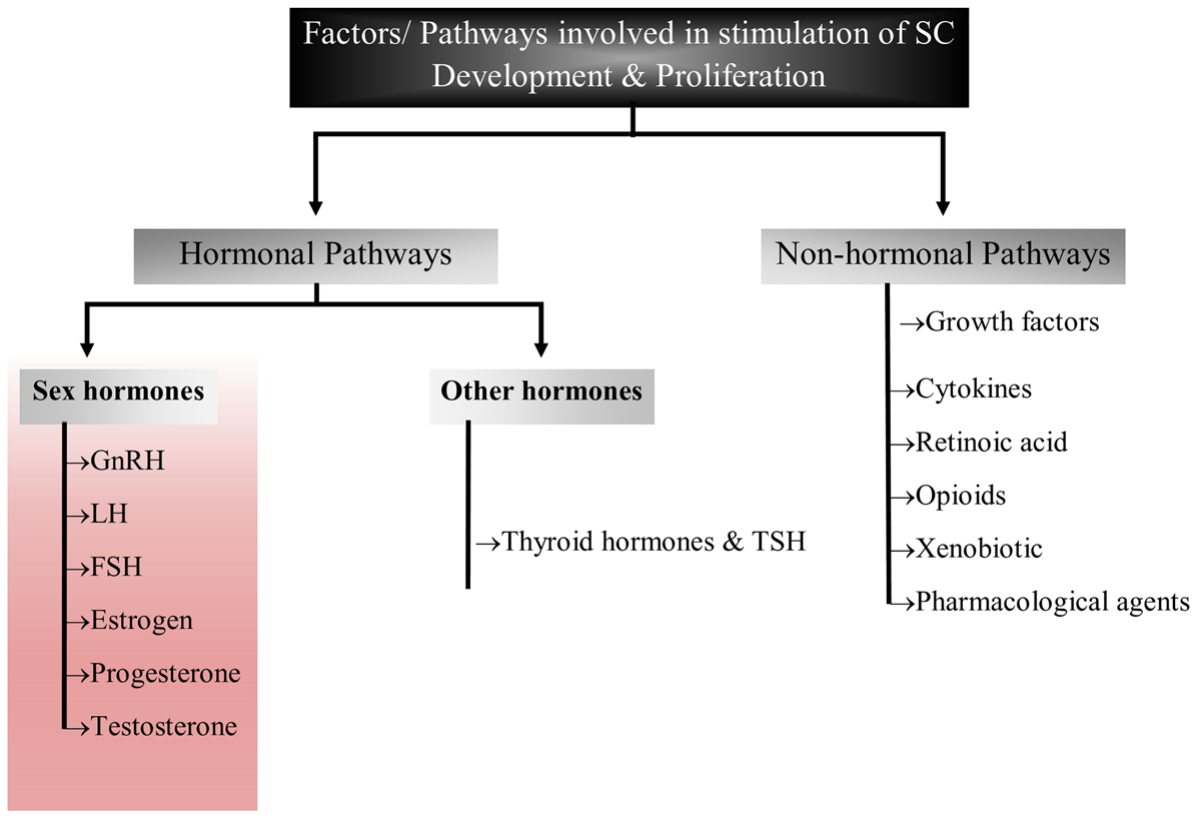
Flow chart diagram representing the factors/pathways involved in Sertoli cell development and proliferation. This figure summarized the role of sex hormones, hormones other than sex hormones and non-hormonal pathways that have been implicated in Sertoli cell development.

The complex process of reproduction is generally regulated by various factors including autocrine, paracrine, juxtracrine and endocrine environment within the gonads (33). Though these processes are well inter-connected, the major function is performed by sex hormones such as leuitinizing hormone, follicle stimulating hormone and prolactin that orchestrate and coordinate sexual development, sexuality and reproduction (34–36). Sex hormones are also playing key roles in development and maturation of Sertoli cells by modulating either Sertoli cell metabolism or influencing growth signaling pathways (14, 27, 31, 35–38). These hormones also create adequate ionic environment in Sertoli cells which is required for germ cell development. In this review, we have discussed the role of reproductive hormones in association with Sertoli cell development, proliferation and maturation (**Figure 2**).

### Follicle Stimulating Hormone (FSH)

FSH plays a crucial role in fertility as it influences the proliferation of Sertoli cells during perinatal life and also stimulates the production of Sertoli cell derived factors that are required for the development of germ cells and testes (39). FSH, LH, thyroid-stimulating hormone (TSH) and chorionic gonadotropin (hCG), belong to pituitary glycoprotein hormone family and these hormones are known to perform important function during Sertoli cell development, thus, directly or indirectly influencing male reproductive health. These hormones are usually existed in the form of a heterodimer which consists of a α-subunit that has the ability to associate with β-subunit (40).

The mechanism of action in which FSH binds and stimulates membrane receptor belonging to the G protein-coupled receptor (GPCR) superfamily (41). It was noted that FSH receptor (FSHR) presents tissue specificity as it is majorly expressed in granulosa cells (female) and Sertoli cells (male) (42). Furthermore, FSHR has the capability to subordinate with other type of G proteins including Gαi to initiate signaling cascade events that modulate Sertoli cell function. Impaired secretion of FSH due to homozygous mutation in the gene encoding β-subunit leads to bilateral small and soft testicles, androgen deficiency, elevated level of LH in serum, low level of testosterone, as well as azoospermia in human (43, 44). Furthermore, homozygous *FSHR* mutations lead to male infertility in few cases, while the A189V *FSHR* mutation in males is linked with subfertility but not azoospermia (45). Interestingly, *Fshr* knockout mice still had sperm production albeit sperm reduction was observed (46–48).

It is a well-known fact that FSH is the factor necessary for Sertoli cell mitogen which stimulates the expression of various Sertoli cell markers such as c-Myc, Cyclin A2, Cyclin D1, and proliferating cell nuclear antigen (PCNA) (39, 49). Moreover, it has been described that FSH level and FSHR expression become stable after puberty, however, a change has been observed in signaling pathways triggered by FSH during transition of Sertoli cells from proliferation to differentiation stage (50). Consistently, some pathways such as FSH-mediated ERK activation and calcium uptake are exclusively activated in immature Sertoli cells during proliferative phase. The opposite action of FSH in immature and mature Sertoli cells is related to the cAMP kinetics (51). It was found that cAMP level was low in immature rat Sertoli cells. On the other hand, higher basal concentration of cAMP was observed in 20 days old Sertoli cells along with almost 4-fold increased activity of phosphodiesterase and completely abolished in older rat Sertoli cells (52–55). Hence, it is assumed that diverse function of Sertoli cells in response to FSH might be responsible for robust onset of germ cell differentiation during prepubertal testicular maturation in rats. What is more, *Gαs* and *Ric8b*, which activate adenylate cyclase for supplementing cAMP production and gene transcription, can also cause constrained FSH action during infancy in primates (56). Thus, the FSH action on Sertoli cell development and maturation is complicated and it is still difficult to investigate the complete array of signaling events *in vivo*.

In fact, it is hard to differentiate the overlap signaling pathways *in vivo* that are triggered during Sertoli cell proliferation and maturation. Most of the studies are conducted *in vitro* and these studies have demonstrated some of the major signaling pathways that are stimulated by FSH. In this regard, a study described that FSH binds with its receptor (FSHR) to form Gα protein, which is further dissociated into two heteromeric molecules, Gα-subunit and Gβ/γ unit. This dissociation further stimulates a cascade signaling mechanism by activating mitogen-activated protein kinase (MAPK), or phosphoinositide 3-kinase (PI3K)/protein kinase B (PKB) and adenylate cyclase/cyclic adenosine monophosphate (cAMP)/protein kinase A (PKA) which cause a change of Sertoli cell membrane potential and calcium influx. During this process, each subunit of FSH heterodimer protein is destined to perform specific function such as Gα subunit is responsible for the activation of adenylate cyclase which further initiates the formation of cAMP and phosphorylation of PKA (57, 58). Furthermore, PKA activates structural proteins, transcription factors and enzymes which trigger diverse biological processes with varying effects on Sertoli cells (37). More specifically, FSH has biphasic effects on membrane potential of immature rat Sertoli cells, which are manifested by membrane hyperpolarization (59).

**Figure 3:**
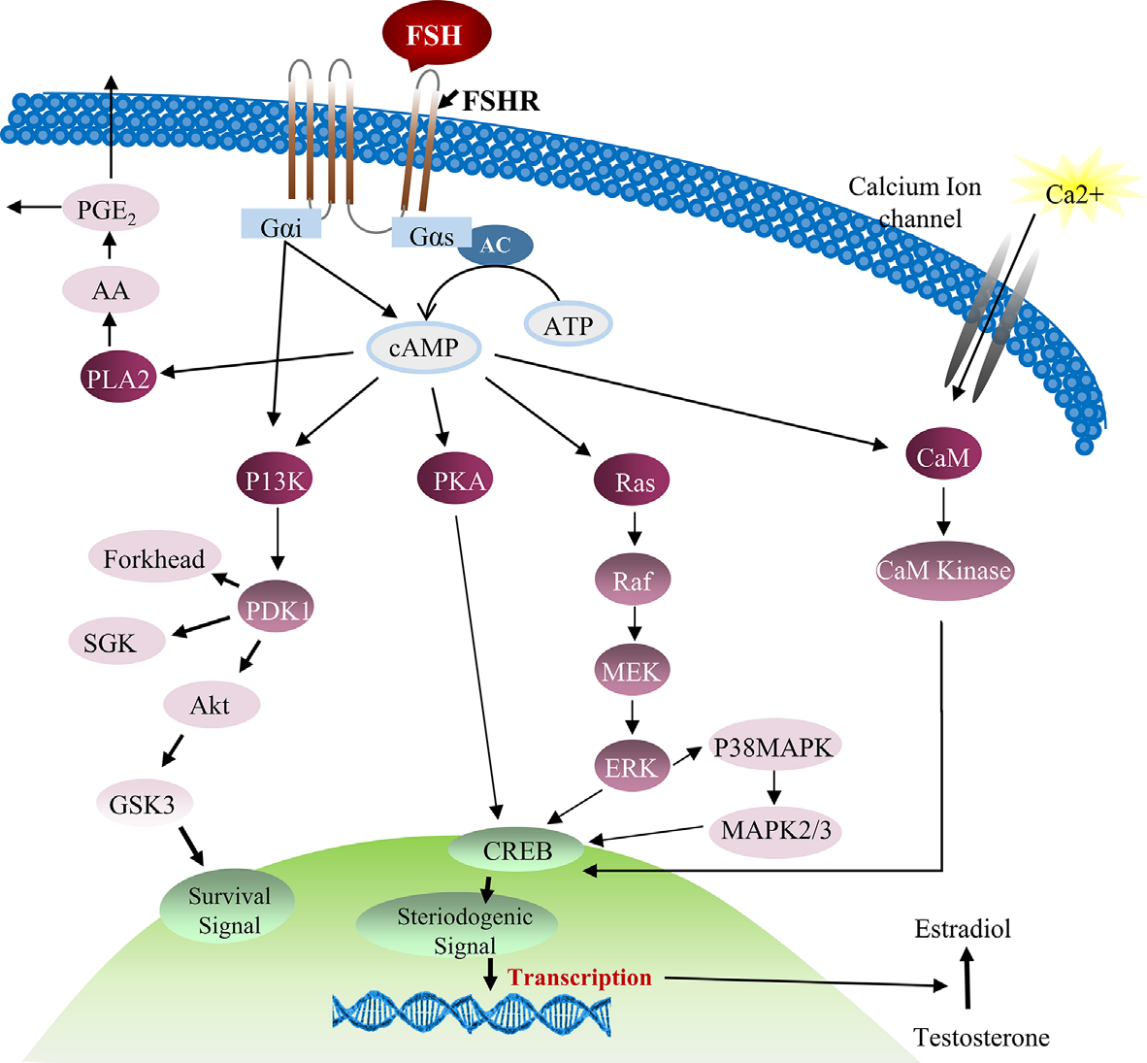
FSH and testosterone signaling pathways in Sertoli cell proliferation. Initially FSH binding to the FSH receptor causes receptor coupled G proteins to activate adenylate cyclase (AC) and increase intracellular cAMP levels. Multiple factors can be activated by cAMP in Sertoli cells including PKA that can phosphorylate a number of proteins and also regulate the expression and activity of numerous transcription factors including CREB. FSH also causes Ca^2+^ influx into Sertoli cells that is mediated by cAMP and perhaps PKA modification of surface Ca^2+^ channels. Depolarization of the cell is also involved in Ca^2+^ influx. Elevated Ca^2+^ levels can activate calmodulin and CaM kinases that have multiple potential downstream effects including the phosphorylation of CREB. During puberty, FSH activates the MAP kinase cascade and ERK kinase in Sertoli cells most likely via cAMP interactions with guanine nucleotide exchange factors (GEFs) and activation of Ras-like G proteins. ERK is capable of activating transcription factors including SRF, c-jun and CREB. FSH and cAMP likely act through GEFs to activate PI3-K and then phosphoinositide dependent protein kinase (PDK1) and PKB in Sertoli cells. FSH also mediates the induction of PLA_2_ and the subsequent release of arachadonic acid (71, 72).

FSH was also found to stimulate cAMP/PKA which intercedes various protein phosphorylation to trigger calcium channels and their regulators. But the complete scenario of FSH stimulation of cAMP/PKA and subsequent voltage gated calcium channels (VDCC) modulation is still not clear. Previous reports described that PKA system phosphorylates α1-subunit of the VDCC resulting in calcium potentiation (60, 61). However, up till now, no research has been conducted to investigate this mechanism in Sertoli cells. The addition of PKA and adenylate cyclase inhibitors [MDL, (Bu)2cAMP and staurosporine] in cultured Sertoli cells can partially impede FSH mediated calcium uptake, indicating involvement of other mechanisms in calcium influx during Sertoli cell proliferation (62). Further evidence showed that Sertoli cell proliferation is not only depend upon AC/cAMP/PKA pathway, some alternative mechanisms also exist, such as FSH-mediated dissociation of the Gαi-GGβ/γ heterodimer which causes calcium influx through L-type VDCC and [^14^C]-MeAIB transport system (63, 64). Moreover, FSH has the ability to transport small amino acids through activation of system A (which is basically designed for the transport of neutral amino acids with small side chains such as alanine, serine and glutamine). System A activation by FSH can provide nitrogen from alanine and other amino acids for biosynthesis of proteins and nucleotides which are essential for cellular growth (65, 66). Similarly, alanine is converted into pyruvate and is used as energy substrate by Sertoli cells. The presence of this alternative mechanism of Sertoli cell proliferation has been validated by inhibition of [^14^C]-MeAIB transport system (67). FSH activates PI3K downstream target, PKB, which further stimulates enhanced uptake of glucose, calcium and small amino acids in cultured Sertoli cells (68). The active PI3K/AKT signaling pathway is required to stimulate the actions of FSH, whereas an active ERK/MAPK pathway can inhibit the expression of aromatase (such as Cyp19a1) (69). Altogether, these pathways are essential for proliferation and differentiation of immature Sertoli cells that pave the way for successful spermatogenesis (70). Taking consideration of all these studies, a comprehensive diagram explaining the role of FSH and other factors in Sertoli cell proliferation can be proposed (27) (**Figure 3**).

### Androgens

Cessation of proliferative phase of Sertoli cells is mediated by changes in gene expression and establishment of BTB and finally Sertoli cells become able to sustain developing germ cells. Thus, it is imperative to investigate the factors that are involved in transition of Sertoli cells from proliferation to maturation phase. In this regard, some studies have demonstrated that androgens and their derivative products are key mediators for Sertoli cell proliferative phase cessation in diverse species (73, 74). In fact, androgens play important functions that reach far beyond the reproductive process, for example 5α-dihydrotestosterone (DHT) regulates glucose consumption and lactate production in cultured rat Sertoli cells (35, 75). Similarly, it is also reported that long time treatment of DHT in cultured human Sertoli cells can cause decrease expression of lactate dehydrogenase A and monocarboxylate transporter 4 (MCT4) levels (76).

The function of androgens is intensively investigated in terms of fertility and spermatogenesis while its role in Sertoli cell maturation and development generally remains elusive, instead of knowing that high amounts of androgens is produced by Leydig cells in the form of testosterone. The dynamic level of testosterone is observed during different developmental stages of organism such as its concentration increases at the end of fetal life, and starts to decrease from birth until puberty, and then increases again (77, 78). Testosterone performs its function through classical and non-classical mechanism. Non-genomic signaling of testosterone can activate gene transcription through CREB mediated pathway (79, 80). Furthermore, zinc transporter ZIP9 subfamily protein that is localized on the plasma membrane also has ability to mediate testosterone level (81).

Testosterone can also function in a non-classical pathway through the androgen receptor to rapidly activate kinases. For example, by increasing testosterone levels, the MAP kinase cascade is rapidly activated in Sertoli cells. An inhibitor of non-classical testosterone signaling blocked meiosis in pubertal mice and caused germ cell loss in adult mouse testes, while a classical pathway inhibitor caused the premature release of immature germ cells. Thus, classical and nonclassical testosterone signaling have overlapping and distinct functions that are required for the spermatogenesis and male fertility. Furthermore, some findings suggested that the non-classical testosterone signaling can act *via* Src and ERK kinases to facilitate the adhesion of germ cells to Sertoli cells (82, 83). On the other hand, the non-classical signaling of androgens alter the cellular process within seconds to minutes (84). This system can increase calcium influx by activation of phospholipase C which causes hydrolysis of phosphatidylinositol 4,5-biphosphate (PIP2) (85). The absence of PIP2 decreases negative charges on membranes and causes closing of K^+^ ATP channels and opening of the VDCC, which results in calcium influx (86). Similarly, the calcium influx stirred by testosterone may be involved in several other biological processes such as cytoskeleton rearrangement, gene transcription and cell proliferation (27, 42). Thus, it is believed that non-classical action of androgens is more closely related to Sertoli cell maturation and spermatogenesis.

Studies that *in vivo* treatment of testosterone caused reduced incorporation of [H^3^] thymidine by Sertoli cells in some species suggesting that testosterone can inhibit the proliferation of Sertoli cells (35, 87). Further work by Buzzard and colleagues displayed that the addition of testosterone in cultured Sertoli cells leads to strong inhibition of proliferation as well as increased expression of cell cycle inhibitor markers such as p27Kip1 and p21Cip1, while it also induces the enhanced expression of GATA-1 which is a marker for Sertoli cell differentiation (88). By crossing hypogonadal (hpg) mice that lack gonadotrophins and intratesticular androgen with mice lacking ubiquitous AR (ARKO) or specifically in Sertoli cells (SCARKO), O’Shaughnessy et al. found that dihydrotestosterone has no effect on germ cell numbers in hpgSCARKO and hpgARKO mice, while testosterone increased germ cell numbers in hpgSCARKO and hpgARKO mice, and this was associated with stimulation of FSH release (89). Thus, androgen stimulation of spermatogenesis requires direct androgen action on the Sertoli cells. However, some studies on mouse model demonstrated controversial results related to androgens involvement in Sertoli cell proliferation. For example, *Tfm* mutant mice lacking functional androgen receptor and *AR* knockout mice displayed reduced Sertoli cell number (90–92). But the observed phenotypes of *Tfm* and *AR* knockout mice could not be attributed entirely to the androgen effect on Sertoli cells since androgens are also known to be produced by peritubular cells. Nevertheless, specific deletion of *AR* in mouse did not show any aberration in Sertoli cell number as well as the expression of Sertoli cell maturation markers (90). These results demonstrated that androgens may affect Sertoli cell proliferation through an indirect way because peritubular cells secrete Activin A which also influences Sertoli cell physiology (93–95). Furthermore, SCAR KO mice showed minor changes which further suggests that the effect of androgen on number of Sertoli cells is not regulated by the direct action. Subsequently, *TgSCAR* (transgenic mouse with gain of function) mice showed reduced Sertoli cell proliferation which further lead to decreased testis size (96). Altogether, it can be deduced that AR expression in Sertoli cells is wisely orchestrated to avoid early maturation of Sertoli cells.

The synergistic actions of testosterone and FSH *via* testicular Sertoli cells regulate male fertility (53). FSH acts through receptors (FSHR) on the Sertoli cell to stimulate spermatogenesis while androgens promote testis growth through receptors (AR) on the Sertoli cells, Leydig cells and peritubular myoid cells. By examining the effects on testis development of ablating FSHRs (FSHRKO mice) and/or ARs ubiquitously (ARKO mice) or specifically on the Sertoli cells (SCARKO mice), results showed that development of most testicular parameters is more dependent on FSH action than androgen action mediated through the Sertoli cells prior to puberty. Post-pubertally, germ cell numbers and spermatogenesis are dependent on FSH and androgen action through the Sertoli cells (91). Furthermore, through the analysis of mice lacking both FSH receptors and androgen receptors in Sertoli cells (FSHRKO-SCARKO), Abel et al. found that FSH and androgen act through redundant, additive, and synergistic regulation in spermatogenesis and Sertoli cell activity (97). Additionally, in pubertal primate (*Macaca mulatta*) Sertoli cells, prolonged stimulation of testosterone significantly elevated the expression of genes involved in FSH signaling pathway such as FSHR, GNAS and RIC8B, and this was associated with a rise in cAMP production. Testosterone also augmented FSH induced expression of genes like SCF, GDNF, ABP and Transferrin. Such a coordinated network of hormonal signaling in Sertoli cells may facilitate the timely onset of the first spermatogenic wave in pubertal primates and is responsible for normal spermatogenesis (53). On the other hand, it has been reported that insufficient FSH and androgen are associated with azoospermia in infantile primate testes (98). Thus, it is assumed that infant primate Sertoli cells may have insufficient number of AR and the binding ability of testosterone to AR might be compromised during primate infancy.

### Luteinizing Hormone

Luteinizing hormone (LH) belongs to the family of glycoproteins, with α subunit and hormone-specific β subunit. LH and FSH both were isolated as molecules in 1942 and these two gonadotrophins are involved in synthesis of estradiol and ultimately form the androgens. LH accelerates testosterone production in Leydig cells, thus, helping in spermatogenesis by directly impacting on Sertoli cells. Knockout mice for LH receptor (*Lhr*) have no testosterone production with disrupted spermatogenesis. This LH-dependent testosterone absence leads to azoospermia, however, in some cases absence of LH signaling does not disrupt the pathway fully and results in oligozoospermia with low testosterone production (99). But knockout mice for luteinizing hormone/choriogonadotropin receptor *(Lhcgr*) had elevated levels of *Wnt5a* (wingless-type MMTV integration site family member 5A) in Sertoli cells that favors cell proliferation. It was also noted that absence of LH caused alternations in genes associated with Sertoli cell development and proliferation (100, 101).

Three genetically modified mouse model were generated to study the effect of LH on Sertoli cell development by completely or partially reducing its activity. Two distinct strategies were used to generate these mutant models; one with LH-deficient hpg-(hypogonadal) mice to selectively study either pituitary-independent transgenic-(tg) FSH or ligand-independent activated tg FSH receptor (FSHR) expression, and second model used LH receptor (*Lhr*)-deficient mice in which their gonads were isolated to examine endogenous mouse FSH effects on gonad development. Analysis of these models showed subtle differences in gem cell maturation between *tg-hpg* and *Lhr*-null mouse models, indicating that the FSH cannot fully restore Sertoli cell number in absence of LH activity (102). Thus, the synergistic effect of both LH and FSH is important for proper proliferation and development of Sertoli cells.

### Estrogens

Estrogens are steroid pleiotropic hormones, present in ovary and testis. These hormones act by cytosolic estrogen receptors (ERs). Alpha (α) and beta (β) receptors are found in animals while in fishes, ERγ has been discovered as the third type receptor. ERα and ERβ are located in the cell membrane; either as homodimers (ERα-ERα or ERβ-ERβ) or as heterodimers (ERα-ERβ). These hormones play their roles in production, regulation as well as maintenance of concentration of testicular liquid (36).

A study conducted by Royer et al. indicated that estradiol initiates the proliferation of Sertoli cells by activating classical estrogen receptors and G protein-coupled estrogen receptor which further induce a cascade of signaling events through CREB activation (103). It is important to mention that estrogen expression in testis is dynamic and varies from postnatal to adult life. Its concentration increases from 20 days of post-partum and continues to increase till 30 days old while aromatase transcripts has not been detected in adult rat Sertoli cells. However, in adult rats, aromatase expression is noted in Leydig, pachytene spermatocytes and round spermatids (104). It is suggested that Sertoli cells may produce estrogen in immature animals while the source of estrogens in adult animals comes from Leydig or germ cells (105). Hence, complete understanding of physiological effects of estrogens is necessary to investigate its actual function in postnatal testis development.

Studies found that *ERα* knockout mice or rats are infertile while *ERβ* knockout mice or rats have no such abnormalities, which indicates that ERα subunit is essential for fertility and reproduction. This function is evident in 15 days old rat Sertoli cells in which ERα promotes cell proliferation by acting on NF-kB (nuclear factor-kB) in P13K and ERK1/2 (extracellular signal-regulated kinase 1/2) manner and ultimately increasing the levels of Cyclin D1. On the other hand, ERβ promotes cell cycle arrest by interacting with 17β-estradiol (E2) (106).

### Progesterone

Progesterone is a major cholesterol-derivative steroid and is specifically involved in reproduction. The receptors of this hormone are localized in the nucleus and cytoplasm of spermatogenic cells, Sertoli cells and occasionally in the Leydig cells. Structurally, two isoform receptors of this hormone exist; namely PR-A and PR-B and these intracellular proteins belongs to nuclear receptor superfamily of transcription factor (107). High level of progesterone has inhibitory role in spermatogenesis by limiting the production of Leydig cells and Sertoli cells at developmental stage (108). The effect of progesterone was examined by generating progesterone receptor (*PR*) knockout mice. *PR* knockout mice displayed large testis size, increased total sperm contents and increased number of Sertoli cells. On the other hand, synthetic progestins such as levonorgestrel (LNG) in combination with testosterone caused suppression of spermatogenesis and increased germ cell apoptosis (108).

### Prolactin

Prolactin is a type of polypeptide hormone that is involved in wide range of biological functions including lactation, osmoregulation, immune articulation and reproduction (109). Prolactin receptors (PLR) are present on Sertoli cells and prolactin through its receptors mediates proliferation of Sertoli cells (110). Various reports reveled its biological function in reproduction and elevated level of prolactin leads to hypogonadism and male infertility (111). It is highly recognized that prolactin regulates testicular function by two ways either altering pituitary function by inducing LH and FSH production or Leydig cells through modulation of testosterone hormone (1). Furthermore, targetted mutation of prolactin receptor in model organism displayed mild phenotype indicating that prolactin has partial effects on male reproductive health (112).

## Other Regulatory Factors Involved in Sertoli Cell Development and Proliferation

Besides the mentioned hormones, many other factors such as growth factors, cytokines, xenobiotic and pharmacological agents, have been identified and are involved in Sertoli cell development process. Opioids, such as α-melanocyte-stimulating hormone (αMSH), β-endorphin and proopiomelanocortin (POMC), mainly produced in Leydig cells, exert direct paracrine actions on Sertoli cell proliferation (113–115). The *in vitro* exposure of fetal human testis to ibuprofen does not modify the number of Sertoli cells but decreases AMH and SOX9 expression, suggesting a role in Sertoli cell maturation (116).

### Insulin Receptor Signaling Family

The insulin receptor tyrosine kinase family consists of insulin receptor (IR), IGF-1R and insulin related receptor (Irr). These receptors are present in all types of cells in eutherian mammals (117). Mice lacking IR and IGF-1R die within four days after birth due to ketoacidosis and respiratory failure, respectively (118). A lot of studies had investigated the function of these hormones and new insights regarding their involvement in reproductive system (29, 50, 117, 119–121). Recently, a study investigated the *in vivo* function of IR and IGF-1R in which both factors work in a synergistic way to regulate the Sertoli cell number and testis size. Furthermore, the study also described that both receptors and their downstream molecules are critical for the development of male gonads and sexual differentiation (122). Similarly, the insulin-related peptide hormone relaxin *(Rlx*) has also been recognized to perform an essential role in reproduction and it precipitates in the regulation of the cyclic adenosine monophosphate and nitric oxide pathways that are implicated in Sertoli cell proliferation (123).

It has been recognized that insulin is involved in energy metabolism and also regulates cell proliferation and differentiation. Generally, the insulin function is interceded by IR through phosphorylation that further activates classical signaling mechanism involving adaptor protein such as insulin receptor substrate-1 (IRS-1) (28). Different studies have reported the function of insulin in testicular development, in modulating testicular cell function (38, 124, 125), or even influencing HPG axis function (28, 29). Furthermore, the compromised function of insulin is the leading cause of Diabetes Mellitus (DM) which is usually accompanied by aberrant testosterone levels (126). Thus, it can be deduced that insulin could regulate testosterone secretion in human and animal models. A study demonstrated that insulin directly influences Sertoli cell metabolism by affecting amino acid accumulation, glucose transport and lactate production either through the modification of glucose transporter expression or altering important glycolytic enzyme activity (121). Further studies indicated that cultured Sertoli cells can cause reduced lactate production and altered caspase-dependent apoptotic signaling (75, 127). Similarly, it has also been reported that insulin activate calcium-dependent membrane depolarization in immature Sertoli cells, which is mostly induced through IGF-1R activation (120). Altogether, these findings clearly indicate the importance of insulin function in regulating Sertoli cell metabolism which is further manifested by Sertoli cell proliferation.

### Cytokines

Various studies reported that inflammatory cytokines are not only produced by macrophages in response to inflammatory signals but these cytokines are also secreted from Sertoli cells and appear to take part in the regulation of Sertoli cell proliferation (1, 19). For example, interleukin-1, 6 (IL-1 and IL-6) and tumor necrosis factor α (TNF-α) are produced by Sertoli cells and *in vitro* studies demonstrated that all of these cytokines are involved in Sertoli cell metabolism by activating the production of transferrin. Furthermore, it was also noted that only IL-1, neither IL-6 nor TNF-α, enhanced lactate production and secretion during Sertoli cell proliferation (1). Notably, IL-1 activity in Sertoli cells can be specifically neutralized by IL-1α antiserum, implying that IL-1α is the major isoform of IL-1 in Sertoli cells (17). But the underlying pathophysiological mechanism is still not completely understood due to the lack of *in vivo* studies. Interestingly, animal model studies with disrupted interleukin or tumor necrosis factors displayed no obvious alterations in testicular development. Thus, the actual function of these cytokines in relevance to Sertoli cell development is still obscure and needs more investigations.

### Thyroid Hormones

It has been described that thyroid hormones regulate lactate production, glucose transporter type 1 mRNA levels, aromatase activity, Sertoli cell proliferation and other processes of Sertoli cells in various mammalian species (128–130). The involvement of thyroid hormones in establishing the Sertoli cell population have been extensively investigated and results indicated that thyroid hormones can affect Sertoli cell proliferation through direct or indirect ways. Generally, indirect way of thyroid hormone on Sertoli cells is mediated by triiodothyronine (T3) that inhibits FSH production and leads to reduced Sertoli cell proliferation (128). Some studies demonstrated that T3 treatment can reduce Sertoli cell proliferation activity, as well as Sertoli cell proliferation period and Sertoli cell number (88, 128, 131). Similarly, it has been described that T3 also stimulates the maturation of Sertoli cells *in vitro* implying that T3 can terminates Sertoli cell proliferation and favors the terminal maturation of Sertoli cells (132). Thyroid hormones can halt Sertoli cell proliferation by accelerating the accumulation of cell cycle inhibitors p27Kip1 and p21Cip1 (88, 133, 134). To be noted, thyroid hormone, retinoic acid, and testosterone share similar suppressive effects on the rate of Sertoli cell division without any apparent additive effects (88). Another study displayed that *Connexins 43* (*Cx43*) could be an intermediate target of T3 in the inhibition of Sertoli cell proliferation (135). Thus, a balance level of thyroid hormones during early life of development is essential for the terminal differentiation of Sertoli cells.

### WNT and BMP Signaling Pathways

The vertebrate WNT (Wingless-related integration site) family consists of 19 secreted cysteine-rich glycoproteins (136). Though WNT signaling exerts an antagonistic effect on testis-determining pathways in sex determination during the embryonic stage, it promotes sperm maturation in adult epididymis (137). During the development of seminiferous tubules, Wnt/β-catenin can play an important role in the differentiation of Sertoli cells. However, these findings appear to be inconsistent about the influence of Wnt/β-catenin signaling. For example, several studies have shown that β-catenin deletion does not induce aberration in Sertoli cells, but β-catenin stabilization results in immaturity, inadequate differentiation and irregular cellular interaction in Sertoli cells, as well as reduced proliferation and increased apoptosis of germ cells (138–141). Similar findings have also been observed when the Wnt/β-catenin pathway is activated in *APC* (adenomatous polyposis coli-conditional) knockout mice (142). Therefore, the suppression Wnt/β-catenin pathway is required to sustain normal maturation and proliferation of Sertoli cells (143).

Bone morphogenetic proteins (BMPs) and transforming growth factor-beta superfamily (TGF-β) also have pivotal roles in reproductive biology. Their roles have been established by various *in vivo* and *in vitro* studies. BMP2, BMP4, BMP8a and BMP8b are involved in specification of primordial germ cell (PGC), acceleration of spermatogonial proliferation as well as are responsible for adult spermatogenesis *in vivo* (144).

A study found that *Bmp4* was expressed in postnatal days 4 and 7 isolated Sertoli cells implying that *Bmp4* perform important role in early postnatal testis development. In testes, multiple BMP genes are expressed and BMP7 and BMP8 a/b transcripts were specifically found in germ cells at various stages of differentiation (145, 146), thus indicating that these molecules may mediate paracrine interactions which are secreted by Sertoli cells. Furthermore, mice lacking BMP8b had smaller testes, similar type of phenotype was also observed in *BMP8a* null mice (147, 148). A recent study explored the role of Sclerostin domain containing 1 protein (*Sostdc1*) in modulating the Sertoli cell gene expression and its possible outcomes on mouse spermatogenesis. Interestingly, Pradhan et al. found that *Sostdc1* is a negative regulator of spermatogenesis, and found that down regulation of *Sostdc1* during puberty is necessary for quantitatively and qualitatively normal spermatogenesis (149). Thus, it is argued that *Sostdc1* is a dual BMP/Wnt regulator and plays indirect role in mouse spermatogenesis by influencing Sertoli cells.

### Activin and Inhibin

Activins are dimeric glycoproteins, consisting of β subunits and members of TGF-β superfamily. Activins mediate FSH production by a cascade of interacting proteins event (150). Their ability to bind with type II receptor causes phosphorylation of type I receptor, starting a series of phosphorylation of SMAD proteins (SMAD2, SMAD3, SMAD4) which ultimately triggers the transcription of FSHβ encoding gene (150, 151). On the other hand, inhibins and follistatin are considered as antagonists of activins (152). They are also glycoproteins but structurally different from activins. Inhibins compete at binding sites for activins which ultimately affects its activation. Thus, activins, inhibins and follistatin collectively form a complex autocrine network that plays a vital role in fertility. The interruptions of these can cause lower testis size, progressive sterility, delayed fertility as well as other fertility-related issues due to defective Sertoli cell development and proliferation (153, 154).

### Retinoic Acid

Retinoic acid (biologically active component of vitamin A) is a major factor that control the complex process of spermatogenesis and is also important driven force of Sertoli cell development (155). RA induce the initiation of spermatogonia differentiation in the mammals and its activity is generally governed by FSH (156). The functional role of RA was verified by generating vitamin A-deficient (VAD) mice that were infertile due to spermatogonia differentiation arrest at the A_aligned_ stage and treating them with RA results in the complete recovery of spermatogenesis (157).

## Summary

Sex hormonal regulation of Sertoli cell proliferation, differentiation and maturation is an intricate process which requires synergistic effects of these hormones along with the regulatory factors including IGF-1R, insulin, thyroid hormones and cytokines. All these hormones and factors have been implicated in various stages of Sertoli cell development and their balanced action of mechanism is mandatory for ensuring accurate Sertoli cell number, establishment of BTB and maintaining spermatogenesis. Although, recent *in vivo* studies explained the involvement of FSH, androgen, estrogen and IGF-1R to be essential for Sertoli cell development, still the complete scenario of this complex process is unresolved. Thus, it is suggested that there are some additional factors needs to be elucidated in future. Similarly, *in vivo* description of some factors such as TGF-α family members, TGF-β, TNF-α, and IL-1 may shed light on complex process of Sertoli cell proliferation and testis development. Subsequently, the detailed mechanism of action of these hormones might give us insights into a better comprehension of hormonal regulation in Sertoli cell proliferation, as well as provide possible therapeutic molecules for human infertility.

## Author Contributions

RK and XJ conceived the review. WS, BS, AK, and SD collected the information. WS, RK, and XJ wrote the paper. JW, XJ, WL, and RK modified the manuscript. All authors contributed to the article and approved the submitted version.

## Funding

This work was supported by the National Key Research and Developmental Program of China (2018YFC1004700), National Natural Science Foundation of China (31890780 and 82071709), the Open Project Fund from Key Laboratory of Reproduction Regulation of NHC (KF2020-07) and Key Laboratory of Male Reproduction and Genetics of NHC (KF202003), and Natural Science Foundation of Qinghai (2019-HZ-823).

## Conflict of Interest

The authors declare that the research was conducted in the absence of any commercial or financial relationships that could be construed as a potential conflict of interest.
